# Mothers' knowledge and utilization of prevention of mother to child transmission services in northern Tanzania

**DOI:** 10.1186/1758-2652-13-36

**Published:** 2010-09-14

**Authors:** Eli Fjeld Falnes, Thorkild Tylleskär, Marina Manuela de Paoli, Rachel Manongi, Ingunn MS Engebretsen

**Affiliations:** 1 Centre for International Health, University of Bergen, Norway; 2 Fafo Institute for Applied International Studies, Norway; 3 Department of Community Health, Tumaini University, Kilimanjaro Christian Medical College, Moshi, Tanzania

## Abstract

**Background:**

More than 90% of children living with HIV have been infected through mother to child transmission. The aims of our present study were to: (1) assess the utilization of the prevention of mother to child transmission (PMTCT) services in five reproductive and child health clinics in Moshi, northern Tanzania, after the implementation of routine counselling and testing; (2) explore the level of knowledge the postnatal mothers had about PMTCT; and (3) assess the quality of the counselling given.

**Methods:**

This study was conducted in 2007 and 2008 in rural and urban areas of Moshi in the Kilimanjaro region of Tanzania. Mixed methods were used. We interviewed 446 mothers when they brought their four-week-old infants to five reproductive and child health clinics for immunization. On average, the urban clinics included in the study had implemented the programme two years earlier than the rural clinics. We also conducted 13 in-depth interviews with mothers and nurses, four focus group discussions with mothers, and four observations of mothers receiving counselling.

**Results:**

Nearly all mothers (98%) were offered HIV testing, and all who were offered accepted. However, the counselling was hasty with little time for clarifications. Mothers attending urban antenatal clinics tended to be more knowledgeable about PMTCT than the rural attendees. Compared with previous studies in the area, our study found that PMTCT knowledge had increased and the counsellors had greater confidence in their counselling.

**Conclusions:**

Routine counselling and testing for HIV at the antenatal clinics was greatly accepted and included practically every mother in this time period. However, the counselling was suboptimal due to time and resource constraints. We interpret the higher level of PMTCT knowledge among the urban as opposed to the rural attendees as a result of differences in the start up of the PMTCT programme and, thus, programme maturation. After comparison with earlier studies conducted in this setting, we conclude that when the programme has had time to get established, both its acceptance and the understanding of the topics dealt with during the counselling increases.

## Background

More than 90% of the children living with HIV are infected through mother to child transmission (MTCT): during pregnancy, around the time of birth, and through breastfeeding [1,2]. Without specific interventions, the rate of MTCT is approximately 15% to 30% if the mother does not breastfeed the child. With prolonged breastfeeding into the second year of life, the cumulative likelihood of infection can be as high as 45% [1]. In high-income countries, MTCT rates of less than 2% are reported, thanks to routine testing, access to antiretroviral (ARV) therapy and safe use of breast milk substitutes [3,4].

Although there has been an increased coverage of the prevention of mother to child transmission (PMTCT) programme globally [5], there are still many unresolved barriers to the programme, particularly in sub-Saharan Africa. Among the main barriers are low access to and low acceptability of testing [6-9]. As a consequence, guidelines recommend implementation of routine counselling and testing as part of the antenatal care services [10]. Further, several studies have documented poor quality counselling [11-14] and low levels of knowledge about PMTCT among both mothers [5,11,13-16] and counsellors [12]. Inadequate counselling is an important reason for mothers' lack of knowledge about PMTCT [11,13-15], which may impede the use of the service [8,11,14,15].

In Tanzania, the estimated HIV prevalence of pregnant women attending antenatal care in 2007 was 8.2% [17]. The PMTCT programme in Tanzania was piloted in 2000 at five clinics [18], and later expanded throughout the country; at the end of 2008, the national coverage of PMTCT was 65% [19]. The experiences gained in the pilot phase were that there was a high acceptability of testing among pregnant women, but the voluntary opt-in strategy to counselling and testing impeded coverage [18]. The national PMTCT guidelines, issued in 2004 and adhered to during this study, recommend implementation of routine counselling and testing [20]. The infant feeding guidelines included were in accordance with the 2001 guidelines from the World Health Organization (WHO) [21]. Updated national PMTCT guidelines were issued in 2007, and had not been implemented during this study [22].

Before and during the pilot testing phase of PMTCT in Tanzania, four studies were conducted in the Moshi district of the Kilimanjaro region. These studies were conducted at antenatal clinics and explored the mothers' knowledge about PMTCT, their infant feeding intentions, their willingness to test for HIV, and the counsellors' perspectives on the PMTCT programme [23-26]. We set out to explore the same topic at five of the same clinics eight years after PMTCT was introduced and in a setting where all of the clinics included in the study had implemented PMTCT with routine counselling and testing in their antenatal care.

The aims of this study were: (1) to assess the utilization of the PMTCT services, in particular HIV counselling and testing, in five reproductive and child health clinics in Moshi after the implementation of routine counselling and testing; (2) to explore the level of knowledge the postnatal mothers had about PMTCT; and (3) to assess the quality of the counselling given.

## Methods

**Figure 1:**
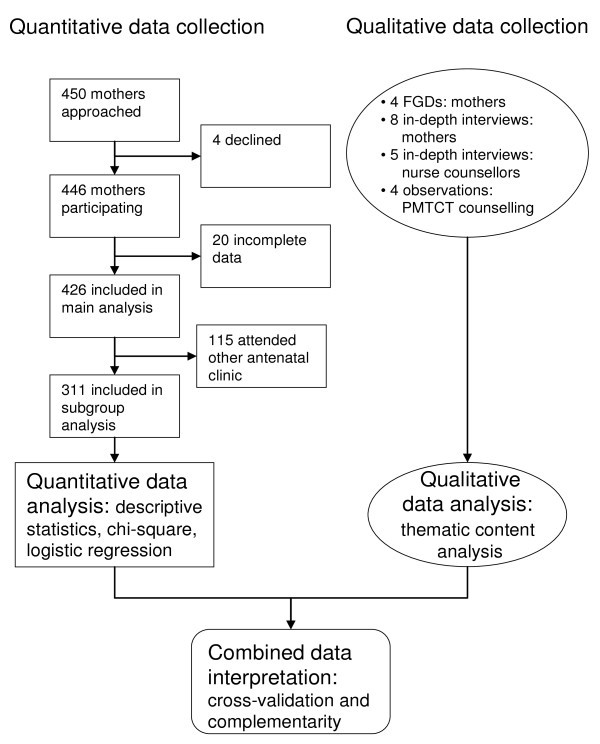
**Mixed methods: concurrent triangulation**.

Mixed methods were used due to the combined exploratory and descriptive research aims (Table 1). We were interested in both the mothers' utilization of the testing and counselling, as well as the experiences of the attending mothers and the employed nurse counsellors at the respective sites. By combining both quantitative and qualitative data, we aimed to cross validate the findings and to reach a greater understanding of the research aims. To achieve this, we used a concurrent triangulation design [27] (Figure 1). A cross-sectional survey was conducted concurrently with qualitative in-depth interviews, focus group discussions and observations at the clinics. The qualitative data served to obtain information from different sources, to provide a broader perspective, and to facilitate the interpretation of the quantitative data. The quantitative and qualitative data were separately analyzed and thereafter integrated during the interpretation of the results.

**Table 1:** Study aims and the quantitative and qualitative methods applied to answer them

Study aim	Quantitative method	Qualitative method	Mixed methods
	Survey of 426 postnatal mothers	4 focus group discussions with mothers	Concurrent triangulation: quantitative and qualitative data were separately collected and analysed. The methods were integrated when interpreting the results.
		8 in-depth interviews with mothers	
		5 in-depth interviews with nurse counsellors	
		4 observations of PMTCT counsellings	
1) Assessment of the utilization of the PMTCT services, in particular HIV counselling and testing, in five reproductive and child health clinics in Moshi after the implementation of routine counselling and testing	Descriptive statistics:	Exploring the mothers':	Quantification of the utilization of the PMTCT service in terms of numbers of mothers counselled and tested
quantitative + qualitative aim	Frequencies of:	Attitudes to the PMTCT programme	And
	Antenatal attendance	Experiences of the programme	Insight into experiences and attitudes to the programme among the mothers and the nurse counsellors (the social and subjective context)
	Received counselling	Barriers to the utilization of the programme	
	Offered test	Exploring the nurse counsellors':	
	Tested	experiences of the mothers acceptance and utilization of the programme	
	Received results	perceived barriers to the programme	
	Urban/rural comparison: Pearson χ^2^		
2) Exploring the level of knowledge the mothers had about PMTCT	Descriptive statistics:	Exploring the mothers':	Quantification of the mother's knowledge on the different questions, compare groups and assess associations
quantitative + qualitative aim	Frequencies of:	Knowledge about PMTCT	And
	Percentage of correct answers to the different questions about PMTCT	misconceptions regarding PMTCT	Validate these findings through a qualitative approach
	Urban/rural comparison: Pearson χ^2^		Reveal and explore misconceptions
	Logistic regression: assessment of factors associated with having little knowledge about PMTCT		
3) Assessment of the quality of the counselling given	Descriptive statistics:	Exploring the mothers':	Quantify numbers of mothers counselled
predominant qualitative aim	Frequencies of:	Experience of and opinions about the counselling received	Indirectly measured by the level of knowledge
	Mothers who had received information on HIV and infant feeding counselling	Understanding of the subjects covered	And
	Indirectly measured by the level of PMTCT knowledge	Exploring the nurse counsellors':	Insight into which subjects the mothers were actually counselled in and which were lacking
		Knowledge about PMTCT	Insight into the knowledge and confidence of the nurse counsellors and their perceived barriers to the counselling
		Perceptions about the counselling given	Insight into the counselling session and the communication during the counselling
		Perception about barriers to the counselling	
		Exploration of the counselling sessions:	
		Subjects covered	
		Level of communication between counsellor and mother	

### Study site

This study was conducted from October 2007 to February 2008 at five governmental clinics in urban and adjacent rural areas of the Moshi district in the Kilimanjaro region in north-eastern Tanzania. HIV testing and counselling were offered on a routine basis in the antenatal care in all of the participating clinics; one of the urban clinics was part of the pilot project of the PMTCT programme in 2000; the other two urban clinics started with PMTCT in 2004, and the two rural clinics implemented the programme in June 2006.

Compared with national data, the Kilimanjaro region has a higher antenatal participation (99% vs. 94%), higher rates of women giving birth in a health facility (70% vs. 47%), a higher level of education (64.9% of the women had completed primary school vs. 50.2%), and a higher literacy rate (91.6% of the women vs. 67%) [28]. In addition, there is higher vaccination coverage: the first dose of diphtheria, pertussis, tetanus and hepatitis B (DPT-HB) and polio immunization at four weeks of age has a coverage of 100% [28].

### Quantitative study population

The sites for the data collection were the same five reproductive and child health clinics that were part of the studies eight years earlier [23-26]. During the data collection period, every mother who came with their infant for first-dose DPT-HB and polio immunization at one of these five clinics was invited to take part in the study. The nurses working at the respective clinics had been thoroughly informed about the purpose of the study. They informed each mother about the study and inquired about her willingness to participate. Individual informed consent in the national language, Swahili, was obtained prior to each interview. In total, 450 mothers were approached, 446 (99.1%) of whom agreed to participate. Of these, 20 were excluded from the data analysis due to incomplete data; the remaining 426 were included (Figure 1).

### Quantitative questionnaire

The questionnaire was translated from English to Swahili by an experienced Swahili teacher, fluent in English, and translated back to confirm wording and meaning. Thereafter, the questionnaire was pre-tested at the five clinics in the study and revised accordingly. Four research assistants, three of them students and the forth a retired nurse who also served as the main research assistant, conducted the interviews. Prior to the start of the study, they were familiarized with the questionnaire and trained in interview techniques by the principal investigator.

The questionnaire consisted of the following: (1) socio-demographic characteristics; (2) information on clinical attendance, birth and infant feeding; (3) PMTCT practice at the clinic (counselling and testing for HIV); and (4) knowledge about PMTCT. Information about HIV status was not collected.

### Quantitative analysis

Data was double entered into EpiData 3.1 software http://www.epidata.dk and analyzed using SPSS PASW. We used descriptive statistics to assess categorical baseline characteristics. Pearson χ^2 ^was used to address potential differences between the urban and rural clinics in terms of population characteristics, PMTCT practice and knowledge. The dependent variable in the crude and adjusted logistic regression analysis was knowledge about PMTCT. The adjusted logistic regression analysis included all the same variables as in the crude analysis. We used the SPSS "backward conditional" command: removal was set at 0.2; and 95% confidence intervals were given.

All but one of the 17 questions about PMTCT knowledge included in our questionnaire were drawn from an already tested questionnaire [29]. Only minor modifications to the questions were made. These 17 questions are presented in Table 2. Eight of the questions were the basis for constructing a knowledge index. In two of the questions (If there are 10 HIV-infected pregnant women, how many do you think would have babies born with HIV? Would you know the number of babies that could get infected through breastfeeding out of 10 HIV-infected mothers?), one to three were classified as correct, while zero and four to 10 were classified as wrong [1]. All other questions had the response options, "yes", "no" and "do not know"; "yes" was scored correct. Every question was weighted equally; one correct answer gave one point. Using the mean as a cut point, those who had zero to five correct answers were classified as having little knowledge about PMTCT, whereas those who had six to eight correct answers were classified as having considerable knowledge about PMTCT.

**Table 2:** Percentage of correct answers to the different questions about PMTCT by type of clinic attended

Question		All included	Subgroup analysis ^S^	
		N = 426 (%)	Rural clinic N = 78 (%)	Urban clinic N = 233 (%)
Is it possible that both parents are positive and the newborn negative? ^i^		363 (85.2)	62 (79.5)	203 (87.1)
When can HIV be passed from mother to child?	During pregnancy ^i^	262 (61.5)	23 (29.5)	163 (70.0)***
	During labour ^i^	414 (97.2)	78 (100.0)	229 (98.3)
	Through breastfeeding ^i^	425 (99.8)	78 (100.0)	233 (100.0)
	Sexual intercourse	262 (61.5)	19 (24.4)	170 (73.0)***
If there are 10 HIV-infected pregnant women, how many babies can be born with HIV? ^i^	1-3	78 (18.3)	13 (16.7)	41 (17.6)
Would you know the number of babies that could get infected through breastfeeding out of 10 HIV-infected mothers? ^i^	1-3	161 (37.8)	12 (15.4)	109 (46.8)***
Can a mother do anything to reduce the risk of transmission to her child during pregnancy? ^i^		350 (82.2)	60 (76.9)	202 (86.7)
If yes, what can she do?	Take medicine	344 (80.8)	58 (74.4)	201 (86.3)
	Use condom	232 (54.5)	10 (12.8)	161 (69.1)***
Can an HIV-infected mother do anything to reduce the risk of transmission to her child during the breastfeeding period? ^i^		305 (71.6)	31 (39.7)	193 (82.8)***
If yes, what can she do?	EBF	215 (50.5)	14 (17.9)	145 (62.2)***
	Use condom	159 (37.3)	2 (2.6)	113 (48.5)***
	Formula milk	304 (71.4)	31 (39.7)	192 (82.4)***
	Cow's milk	303 (71.1)	29 (37.2)	193 (82.8)***
	Breast care	261 (61.3)	19 (24.4)	174 (74.7)***
	Oral thrush	265 (62.2)	18 (23.1)	177 (76.0)***

^S ^Subgroup analysis (n = 311) of rural and urban clinic does not add up

^i ^Included in the PMTCT knowledge index

* p < 0.05

** p < 0.01

*** p < 0.001

Socio-economic status was assessed by constructing an index using principal component analysis (PCA), commonly used when creating socio-economic indices in low-income settings [30]. PCA is a "data reduction" technique that transforms a number of possibly correlated variables (here, socio-economic variables) into a smaller number of uncorrelated variables called principal components. The following background variables were included in our model: (1) the number of rooms and beds in the household and the number of people living in the household per room and per bed; (2) type of toilet, source of fuel for light and cooking; (3) assets (TV, refrigerator, sofa, cupboard, mobile phone); (4) building material (floor and walls); (5) number of chickens, goats, pigs and cows owned; and (6) use of land for farming, and whether the household had purchased seeds or fertilizer the previous year. The first principal component, which is expected to explain wealth, explained 44.8% of the variance in our model. Socio-economic quintiles were constructed based on an index derived from the first component.

Among the included mothers, approximately one-quarter had antenatal attendance at a clinic other than one of the recruitment clinics where they came for immunization (Figure 1). Since we were interested in antenatal practices and were unable to collect comprehensive information of all these other antenatal clinics, we did a sub-group analysis including only the participants who had antenatal attendance at one of the five recruitment clinics. In this analysis, we explored whether there were any differences in PMTCT practice and PMTCT knowledge between mothers who had antenatal attendance at the urban as opposed to the rural recruitment clinics.

### Qualitative data

We conducted eight in-depth interviews with mothers: three with mothers coming to one of the recruitment clinics for DPT-HB and polio immunization, and five with mothers with a child less than one year old. The aim of the in-depth interviews was to elaborate on questions asked in the survey so as to gain a deeper insight and get answers not easily obtained from surveys.

In addition, we carried out four focus group discussions (FGDs) with mothers. By employing FGDs, we aimed to make use of group interactions, which may help people to explore and clarify their views in a way that would be less accessible than in one-to-one interviews [31]. One of the FGDs had 12 participants, while the other three FGDs each had nine participants.

The mothers coming for immunization were approached at the clinic by the main research assistant and the principal investigator and asked if they were willing to participate. The mothers included in the in-depth interviews and the FGDs were recruited in different communities in urban and rural settings of Moshi, assisted by the main research assistant's acquaintances and village leaders. The recruitment criterion was having a child less than one year. Thus, the mothers were purposively chosen on the basis of having been exposed to PMTCT activities within reasonable time.

We also carried out five in-depth interviews with nurse counsellors, one in each of the recruitment clinics. They were approached by the principal investigator and asked if they were willing to participate. Finally, we observed a total of four PMTCT pre- and post-test counselling sessions at three of the recruitment clinics. In one of the urban clinics, we were not permitted to observe the counselling sessions, while in one of the rural clinics, we did not succeed in doing so. The observations were made after having received consent from the nurse counsellor and the mother being counselled. Individual informed consent was obtained from all of the participants in the in-depth interviews and the FGDs.

A semi-structured interview guide was prepared specifically for each group of informants. Themes included were experiences of the PMTCT programme, mothers' knowledge about PMTCT, and perceived barriers to PMTCT. The mothers who came for DPT-HB and polio immunization and the nurse counsellors were interviewed at the clinics, whereas the mothers with a child less than one year old were interviewed in their private homes. The FGDs were conducted outdoors, in a private home or in a church.

All of the in-depth interviews were carried out by the principal investigator (EFF). The interviews with the nurse counsellors were performed in English, while the interviews with the mothers were performed using the main research assistant as an interpreter. She was fluent in English and Swahili, as well as the main local languages. The FGDs were moderated by a nurse working at a local HIV organization. She had training and experience in conducting FGDs. The discussions were all conducted in Swahili. The FGDs and the in-depth interviews ranged in length from 45 to 90 minutes. The in-depth interviews, the FGDs and the observations at the clinics were all tape recorded and subsequently transcribed verbatim. Interviews conducted in Swahili were then translated into English.

Qualitative data analysis was primarily performed by the principal investigator using a thematic content approach [31]. The information in each interview was summarized and grouped according to the information categories in the semi-structured interview guides. Illustrative quotations were selected. During this process, new categories emerged and were added to the analysis, e.g., misconceptions about transmission routes.

### Ethics

The study obtained research clearance from National Institute for Medical Research Tanzania, the Tanzanian Commission for Science and Technology, the Kilimanjaro Christian Medical College Ethical Research Committee, and the Regional Committees for Medical and Health Research Ethics for Region West, Norway.

## Results

### Sample characteristics

The median age of the 426 mothers was 25 years, and the median age of the infants was four weeks. Nearly half of the respondents reported that they lived in rural areas (Table 3). The majority (90.1%) of the mothers were married or cohabiting. Almost half (43.7%) of the respondents were Catholic. The most common ethnic group was Chagga (62.4%). Five of the mothers had never been to school, 49.8% had completed primary school, and nearly half (44.9%) had a secondary or higher education.

**Table 3:** Baseline characteristics of the 426 surveyed mothers by type of clinic attended

Background factor	All included	**Subgroup analysis**^**S**^	
	N = 426 (%)	Rural clinic N = 78 (%)	Urban clinic N = 233 (%)
Residence			
Rural	193 (45.3)	76 (97.4)	50 (21.5)
Urban	233 (54.7)	2 (2.6)	183 (78.5)***
Mothers' age, y			
< = 25	219 (51.4)	45 (57.7)	110 (47.2)
>25	207 (48.6)	33 (42.3)	123 (52.8)
Number of siblings			
0	169 (39.7)	34 (43.6)	79 (33.9)
1	132 (31.0)	20 (25.6)	80 (34.3)
< = 2	125 (29.3)	24 (30.8)	74 (31.8)
Marital status			
Married/cohabiting	384 (90.1)	67 (85.9)	213 (91.4)
Single/divorced/widow	42 (9.9)	11 (14.1)	20 (8.6)
Religion			
Catholic	186 (43.7)	49 (62.8)	92 (39.5)
Protestant	162 (38.0)	25 (32.1)	93 (39.9)
Muslim/other	78 (18.3)	4 (5.1)	48 (20.6)**
Ethnicity			
Chagga	266 (62.4)	66 (84.6)	135 (57.9)
Pare/other	160 (37.6)	12 (15.4)	98 (42.1)***
Education, mother			
0-6	23 (5.4)	5 (6.4)	9 (3.9)
7	212 (49.8)	45 (57.7)	113 (48.5)
8-12	146 (34.3)	21 (26.9)	83 (35.6)
12+	45 (10.6)	7 (9.0)	28 (12.0)
Socio-economic status			
Bottom quintile	81 (19.0)	28 (35.9)	27 (11.6)***
2^nd ^quintile	88 (20.7)	22 (28.2)	41 (17.6)
3^rd ^quintile	94 (22.1)	17 (21.8)	56 (24.0)
4^th ^quintile	65 (15.3)	8 (10.3)	41 (17.6)
Top quintile	98 (23.0)	3 (3.8)	68 (29.2)

^S ^Subgroup analysis (n = 311) of rural and urban clinic does not add up

* p < 0.05

** p < 0.01

*** p < 0.001

The sub-group analysis included 311 (72.9%) mothers, of whom 233 (74.9%) had attended antenatal care at one of the three urban clinics included in the study and 78 (25.1%) had attended one of the two rural clinics. We found significant differences (p < 0.001) between the mothers in the following areas: mothers who went to an urban clinic were more often Muslim, less often Chagga and usually wealthier than those who went to a rural clinic.

### Antenatal clinical attendance

All the 426 mothers had attended the antenatal clinic during their most recent pregnancy. The median number of visits was four (range 1-10). Relatively few mothers (17.8%) reported visiting the antenatal clinic during their first trimesters; the majority (69.0%) presented themselves during their second trimesters. The vast majority of the mothers (85.7%) had given birth at a hospital, a small minority (13.1%) at a health post, and only 1.2% at home or during transport.

In the sub-group analysis, we found that the rural antenatal attendees were more likely to present themselves at the antenatal clinic as late as in the third trimester (29.5%) than the urban attendees (8.2%) (p < 0.001).

### Routine counselling and testing

The majority of the 426 mothers were familiar with the PMTCT programme at the antenatal clinics (Table 4). Information about HIV had been given to nearly all mothers (94.6%) during antenatal care, and two-thirds (65.5%) reported having received infant feeding counselling. There was an almost complete coverage of HIV testing: 97.7% of the mothers had been offered an HIV test, all of them had accepted being tested, and only one of them had not received her results.

**Table 4:** PMTCT practice of the 428 surveyed mothers by type of clinic attended

Practice	All included	**Subgroup analysis**^**S**^	
	N = 426 (%)	Rural clinic N = 78 (%)	Urban clinic N = 233 (%)
Heard about PMTCT programme	394 (92.5)	71 (91.0)	221 (94.8)
Received infant feeding counselling	279 (65.5)	47 (60.3)	169 (72.5)
Received information about HIV	403 (94.6)	75 (96.2)	226 (97.0)
Offered HIV test	416 (97.7)	78 (100.0)	232 (99.6)
Did test	416 (97.7)	78 (100.0)	232 (99.6)
Received results	415 (97.4)	78 (100.0)	231 (99.1)

^S ^Subgroup analysis (n = 311) of rural and urban clinic does not add up

* p < 0.05

In the sub-group analysis, we did not find any significant (p < 0.05) difference between the urban and rural antenatal attendees with regards to PMTCT practices, i.e., receiving counselling and testing (Table 4).

The qualitative data generally confirmed the quantitative findings. The mothers had a favourable view of the PMTCT programme at the clinics and were informed about its content. They seemed to be aware that testing for HIV was part of the antenatal service before arriving at the clinics, and the majority stated that they had discussed it with their partners before attending. Testing was perceived as purely beneficial, both in terms of knowing their own health status and being able to protect their children from infection. No objections to testing were raised by the mothers who were interviewed. The nurse counsellors focused on each mother's opportunity to reject testing, but had never experienced a mother refusing to be tested for HIV. According to the nurses, the mothers were prepared to test when they arrived at the antenatal clinics. Further, the nurse counsellors explained the high acceptability with the fact that the mothers were aware of the benefits that an HIV-infected mother would receive:

The mothers agree to be tested because they know that after they have been tested and found to be HIV-infected, they will get drugs to prevent the infection from mother to the foetus. (Nurse counsellor # 3, rural)

Most clinics had group information about PMTCT for the antenatal mothers, followed by individual pre- and post-test counselling. Although the nurse counsellors seemed knowledgeable in PMTCT, several of the mothers stated that they had received insufficient information during the counselling. During the observations of the PMTCT counselling, we noticed that two of the nurse counsellors gave cursory counselling. In the other two observations, the mothers were given comprehensive information, covering the main areas of PMTCT, except for infant feeding. Due to time constraints, the information was given hastily and the mothers had little opportunity to interrupt with questions if they did not understand. The nurse counsellors were well aware of this potential quality constraint:

We have a lot of clients and few nurses, so the counselling will sometimes not be quite good. (Nurse counsellor # 4, urban)

During the interviews with the nurse counsellors and the observations of the PMTCT counsellings, we did not find any differences between the urban and rural antenatal clinics in the quality of the counselling being provided.

### PMTCT knowledge

The 426 mothers were well informed of the risk of MTCT of HIV through breastfeeding (99.8%) and during labour (97.2%), but only 61.5% knew that it could be transmitted during pregnancy (Table 2). In general, the mothers overestimated the risk of infection. The majority of the mothers knew that it was possible to reduce the risk of transmission during pregnancy (82.2%) and the breastfeeding period (71.6%). However, knowledge of the preventive effect of condoms had not reached all the mothers; 54.5% confirmed it as a preventive during pregnancy and 37.3% during the breastfeeding period. Further, only half of the mothers knew that exclusive breastfeeding would reduce the risk of transmission during the breastfeeding period.

**Figure 2:**
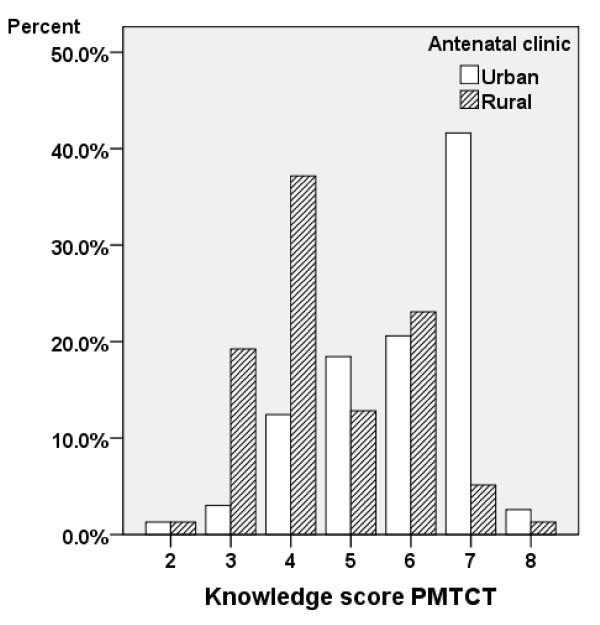
**Knowledge score PMTCT by type of clinic attended**.

There were significant differences (p < 0.05) between the mothers attending antenatal care at the rural and the urban clinics: the urban attendees were more knowledgeable in nearly all subjects. Overall, the median number of correct answers was 12 out of 17. The urban attendees had a median score of 14 and the rural attendees had a median score of 5.5. The knowledge index had a Cronbach's alpha of 0.598. The median number of correct answers to the eight questions included in the knowledge index was six for the urban attendees and four for the rural attendees (Figure 2). Thus, 35.2% of the urban attendees and 70.5% of the rural attendees were classified as having low knowledge scores.

In the adjusted logistic regression analysis, the following factors were associated with having little knowledge about PMTCT (Table 5): (1) the mother was older than age 25; (2) the infant had none or more than one sibling; (3) the mother was non-Christian; (4) the mother presented herself at the antenatal clinic late in the pregnancy; (5) the mother had not received infant feeding counselling; and (6) the mother had attended a rural antenatal clinic.

**Table 5:** Odds ratio of little knowledge about PMTCT for all the 426 surveyed mothers

Background factor	N = 426 (%)	Little knowledge	OR (95% CI)	AOR (95% CI)
		PMTCT N (%)		
Mothers' age, y				
< = 25	219 (51.4)	89 (40.6)	1	1
>25	207 (48.6)	102 (49.3)	1.419 (0.967-2.082)	1.842 (1.119-3.032)*
Number of siblings				
0	169 (39.7)	85 (50.3)	1	1
1	132 (31.0)	43 (32.6)	0.477 (0.298-0.766)**	0.454 (0.266-0.776)**
< = 2	125 (29.3)	63 (50.4)	1.004 (0.632-1.595)	0.654 (0.358-1.193)
Marital status				
Married/cohabiting	384 (90.1)	169 (44.0)	1	
Single/divorced/widow	42 (9.9)	22 (52.4)	1.399 (0.739-2.649)	
Religion				
Christian	350 (81.7)	150 (43.1)	1	1
Muslim/other	78 (18.3)	41 (52.6)	1.463 (0.894-2.394)	1.725 (1.006-2.956)*
Ethnicity				
Chagga	266 (62.4)	127 (47.7)	1	
Pare/other	160 (37.6)	64 (40.0)	0.730 (0.490-1.086)	
Education, y				
0-7	235 (55.2)	105 (44.7)	1	
8+	191 (44.8)	86 (45.0)	1.014 (0.691-1.489)	
Socio-economic status				
Lowest 60%	263 (61.7)	131 (49.8)	1	
Highest 40%	163 (38.3)	60 (36.8)	0.587 (0.394-0.875)**	
Antenatal clinic				
Rural	78 (18.3)	55 (70.5)	1	1
Urban	233 (54.7)	82 (35.2)	0.227 (0.130-0.396)***	0.232 (0.127-0.425)***
Other	115 (27.0)	54 (47.0)	0.370 (0.201-0.681)**	0.298 (0.153-0.578)***
First visit antenatal				
Early (1^st ^and 2^nd ^trimester)	370 (86.9)	153 (41.4)	1	1
Late (3^rd ^trimester)	56 (13.1)	38 (67.9)	2.994 (1.647-5.444)***	2.154 (1.111-4.177)*
Number antenatal visits				
1-2	52 (12.2)	27 (51.9)	1	
3+	374 (87.8)	164 (43.9)	0.723 (0.404-1.293)	
Received infant feeding counselling				
Yes	279 (65.5)	100 (35.8)	1	1
No	149 (34.5)	91 (61.9)	2.909 (1.924-4.397)***	2.303 (1.467-3.616)***
Received HIV information				
Yes	403 (94.6)	175 (43.4)	1	1
No	25 (5.4)	16 (69.6)	2.978 (1.119-7.396)*	1.991 (0.738-5.372)

* p < 0.05

** p < 0.01

*** p < 0.001

As in the quantitative findings, the mothers in the in-depth interviews and the FGDs generally knew about the main transmission routes, but tended to overestimate the risk of transmission, especially through breastfeeding. There was a common misconception among the mothers that the infant was protected in the uterus, and thus could not be infected:

The baby has security in the uterus. (Participant FGD # 2, rural)

Overall, the mothers in the qualitative interviews tended to be knowledgeable about the use of condoms as a preventive measure during both pregnancy and the breastfeeding period. However, several expressed doubts as to whether their partner would accept using condoms, as illustrated in one of the observed PMTCT counselling sessions:

You should also encourage your partner to test for HIV. If you tell him to use condoms during your window period until he has also taken the test, will he agree? (Urban nurse counsellor, observation # 1)

No ... [laughter] he would say I am disrespecting him. (Mother being counselled)

We did not find a difference in the level of knowledge about PMTCT between the urban and the rural mothers in the qualitative interviews.

### Infant feeding counselling

During the observed PMTCT counselling sessions, none of the nurse counsellors talked about infant feeding. Infant feeding counselling appeared to be a priority only for mothers who were HIV infected. The infant feeding options that the nurse counsellors stated that they gave to HIV-infected mothers were in accordance with the 2001 guidelines from WHO [21], namely: exclusive breastfeeding (EBF) for three to six months, formula or cow's milk. Several of the nurse counsellors stated that replacement feeding was a safer option than EBF and did not acknowledge the beneficial effects of EBF in preventing malnutrition and diarrhoea.

However, according to their experience, the majority of the mothers opted for EBF due to their financial situation. In general, the nurse counsellors believed that to exclusively breastfeed for three to four months was more feasible than the recommended six months, and several recommended this duration in the counselling:

Most HIV-infected mothers choose to exclusively breastfeed up to three months, because feeding formula from birth will be too expensive. Even at three months not all can afford to buy milk. (Nurse counsellor # 4, urban)

In the quantitative survey, the mothers were asked the hypothetical question: how would they have fed their infants if they were HIV infected? Half of the mothers (49.5%) stated that they would have given cow's milk, 27.2% would have given formula milk, and 21.8% would have practiced EBF. There was a significant difference (p < 0.001) between the mothers attending the rural and the urban antenatal clinics: the rural attendees were more inclined to give cow's milk (74.4%) and the urban attendees more inclined to give formula milk (32.2%) and to practice EBF (26.6%). The mother's choice of infant feeding if she had been HIV infected was strongly associated with her PMTCT knowledge (p < 0.001). Mothers who would have opted for cow's milk were more likely (60.8%) to have little knowledge about PMTCT, and mothers who would have chosen EBF were less likely (10.8%) to have little knowledge about PMTCT.

The majority of the mothers in the in-depth interviews and the FGDs seemed confused about how HIV-infected mothers should feed their infants. Many questioned the safety of breastfeeding and stated that they would not have breastfed due to the risk of infecting the child:

I will ask a neighbour for cow's milk and boil it rather than use my own milk to avoid the risk of infection. (Participant FGD # 1, urban)

I heard that if you are HIV infected and you breastfeed your baby, your baby will be infected as well, so how can you breastfeed? (Participant FGD # 2, rural)

At the same time, several mothers showed notable knowledge about the protective advantages of EBF and how to reduce the risk of transmission through breast milk:

I would breastfeed the baby for six months without giving anything. The first breast milk is very important to the baby. If you stop, you can never give breast milk again, because if you give alternatives, it will make bruises in the colon which will lead to transmission if it is mixed with breast milk. (Participant FGD # 3, rural)

Make sure she (the HIV-infected mother) has no wounds on her breasts and the baby should not have ulcers in his mouth. (Participant FGD # 4, urban)

## Discussion

In this study in the Kilimanjaro region in Tanzania of urban and rural mothers who had recently been through the PMTCT programme with routine counselling and testing coming for postnatal follow up, we noticed the following: (1) routine counselling and testing was greatly accepted, and nearly all of the mothers had tested for HIV; (2) overall, the mothers were knowledgeable about PMTCT; (3) there were large differences in the PMTCT knowledge of the rural and urban antenatal clinic attendees; (4) the nurse counsellors were generally knowledgeable about PMTCT and had confidence in their own counselling; and (5) the counselling was usually hasty with little time for clarification.

Compared with the previous studies conducted eight years earlier [23-26], we found an impressive improvement in the testing rate, the mothers' PMTCT knowledge and the nurse counsellors' confidence in their own counselling.

### High acceptance of routine counselling and testing

In our study, we found 100% acceptance of HIV testing among the antenatal mothers. In the report from the pilot of the PMTCT programme in Tanzania [18], the voluntary opt-in strategy was identified as a barrier to the testing rates, and in the previous study, only 7% of the pregnant women had tested for HIV [23]. Thus, it seems that the implementation of routine counselling and testing has increased the numbers of mothers testing, as has been demonstrated before [32-35].

It has been suggested that routine counselling and testing increase the acceptance of testing due to a view of it being a part of the "standard of care" offered to all antenatal attendees [32], thus reducing the stigma associated with testing [35]. The acceptance of the testing in our population may also have been facilitated by the widespread knowledge of the benefits of taking part in the PMTCT programme [32]. Furthermore, a mother's prior awareness of testing for HIV being a part of the routine antenatal care seemed to have given her time to discuss it with her partner and prepare for the test before arriving at the clinic.

### Increased PMTCT knowledge

It is difficult to compare the level of knowledge in our study with the level found in other studies because different questions were used. We may have documented a higher overall level of PMTCT knowledge than that found in many previous studies [5,11,13-16], and we claim to see an improvement compared with the studies conducted eight years ago [23]. We interpret this as a result of programme maturation; namely, that the testing rates, the acceptance of the programme and the general knowledge among the participants tend to increase when the programme has had time to get established. Further, these components of the programme are likely to reinforce each other as part of the maturation process.

We did not find any link between levels of education of the mothers and knowledge of PMTCT, which may be due to the generally high and equal level of education in this region. Nor did we find different levels of knowledge among mothers who reported having received HIV information and those who had not, which is probably because nearly all of them had received this information.

In both the quantitative and the qualitative data, we found three main areas where the mothers seemed to have insufficient knowledge about PMTCT: (1) the possibility of MTCT during pregnancy; (2) the protective effect of condom use during pregnancy and the breastfeeding period; and (3) the infant feeding method that is recommended for an HIV-infected mother.

We are unable to explain why so many mothers seemed to be unaware of the risk of transmission during pregnancy. In the previous study eight years ago [23], a much larger percentage (90%) of the mothers were aware of this transmission route. From our qualitative findings, information about this transmission route was given in the counselling; still, many mothers had the misconception that the infant was protected in the uterus. This erroneous belief needs to be addressed and corrected in counselling.

The mothers' apparent lack of knowledge about the importance of condom use during pregnancy and the breastfeeding period may be due to their misinterpretation of the questions (Table 2). The questions address knowledge about avoidance of the primary infection of the mother and avoidance of re-infection of the mother, both of which increase the risk of infecting the child. Furthermore, the use of condoms is a sensitive taboo issue, which may have made it difficult for the mothers to properly answer these questions. In addition, the lack of knowledge may be rooted in the complexity of the issue, implying that the mothers had not fully understood the counselling they received. Thus, it is important that this issue be properly explained during the counselling. One final explanation for the apparent lack of knowledge about condoms may be that the mothers were not empowered to request that their partners use condoms, and condom use was therefore regarded as unattainable. This illustrates the importance of including partners in the PMTCT programme.

### Knowledge gap between mothers attending urban and rural clinics

There were major differences in the PMTCT knowledge of the mothers attending the urban and rural antenatal clinics. Among the factors that were associated with having little knowledge about PMTCT in the adjusted regression analysis (Table 5) was that a larger proportion of the rural attendees presented themselves at the antenatal clinics in their third trimesters compared with the urban attendees. This may explain part of the observed knowledge gap.

In the adjusted analysis, being non-Christian was associated with having less knowledge. However, non-Christians were barely represented in the rural group and thus cannot explain the difference in knowledge between the groups. Further, given that we found that the rural and urban antenatal attendees received similar PMTCT services (Table 4), this does not seem to explain the observed knowledge gap. However, there might be differences in the quality of the counselling received. But the qualitative interviews with the nurse counsellors and the observation of PMTCT practices at both the rural and the urban clinics suggested that the counselling received by the mothers attending the rural clinics was not of inferior quality.

There are many other factors, which are outside the scope of this study, which also could have explained the differences observed, like exposure to other HIV-related education programmes. What we know is that the PMTCT programme was implemented on average two years earlier in the urban clinics than in the rural ones. This may suggest that the differences observed in the PMTCT knowledge of rural and urban mothers could be due to the difference in the maturation of the PMTCT programme: the knowledge had had more time to get established in the population.

However, the observed knowledge gap in the quantitative data between the urban and rural mothers was not found in the qualitative data. There could be several explanations for this. One is that the quantitative data might capture a trend not seen at the individual level. Further, the divergence between the methodologies and the way knowledge was measured may have influenced the difference in results. The qualitative instrument allowed a deeper understanding of the mothers' actual knowledge than the quantitative, more rapid assessments. This illustrates the usefulness of mixing methods that force us to present diverging results.

### The difficult infant feeding counselling

The counselling that the nurse counsellors said they gave to HIV-infected mothers had not been updated to the 2006 WHO infant feeding guidelines [36], which give EBF a stronger stand. Cow's milk was still designated as an infant feeding option, and the recommended duration of EBF was often too short, which is not in accordance with recent knowledge. In two former studies in the Kilimanjaro region, nurse counsellors reported that they had very limited opportunities to keep themselves updated [24,37], which seems to still be the case.

Most nurse counsellors seemed to perceive the risk of HIV transmission through breastfeeding as a greater threat than the risk of malnutrition and diarrhoea if the infant was not breastfed. Nevertheless, the majority of the nurse counsellors considered EBF the best option for most HIV-infected mothers because of their financial situations. Previous studies in the Kilimanjaro region indicated that the nurse counsellors did not have confidence in their own professional knowledge about infant feeding practices for HIV-infected mothers [24,37] and that most of them questioned EBF as a safe alternative [24,37,38]. Thus, both the nurse counsellors' confidence in their knowledge and their attitudes to EBF for HIV-infected mothers seemed to have improved. The fact that there is a lower risk of HIV transmission with EBF than with mixed feeding was a new and unconfirmed finding eight years ago [39], and this may explain part of these differences.

The minority of mothers, who said they would have opted for EBF if, hypothetically, they were HIV-infected, were less likely to have little knowledge about PMTCT. The mothers' infant feeding choices may reflect their overestimation of the likelihood of HIV transmission through breast milk, or a lack of understanding of the advantages of practising EBF. The complexity of the issue and inconsistent infant feeding information may explain the large discrepancies in the level of knowledge about EBF among the mothers. Our findings further underscore the importance of a rapid implementation of WHO's 2010 infant feeding guidelines [40], with increased focus on EBF.

It seemed that the nurse counsellors prioritized giving infant feeding counselling only to the HIV-infected mothers. Due to time constraints, this seems to be a sensible approach. However, it is important that healthy infant feeding practices for both infected and non-infected mothers, with a particular focus on EBF, is covered at the antenatal clinics.

### Increasing workload

Good counselling takes time, and a shortage of staff is a major barrier affecting mothers' PMTCT knowledge. The implementation of PMTCT at the antenatal clinics has increased the staff workload [37,41]. The scale up with implementation of routine counselling and testing is likely to have added to this [32,35,42]. From the PMTCT observations and our interviews with the nurse counsellors, it was evident that the lack of personnel and time often made the counselling hasty and of lower quality than the nurse counsellors would ideally have opted for [13,37]. This could lead to important messages drowning in the information flow, and there was little room for in-depth explanations and clarifications.

We recommend that the nurse counsellors continue to give the majority of the pre-test information in groups to save time, as recommended in the national PMTCT guidelines [20,22]. In subsequent individual counselling, we suggest that the nurse counsellors encourage the mother to repeat the main information received and clarify any misconceptions.

In Tanzania, it is the nurses, already overloaded with tasks, who are trained as counsellors. Experiences from other countries in sub-Saharan Africa have shown that the use of community or peer counsellors to augment counselling capacity is feasible and acceptable [43-45]. Further, this task shifting has been found to increase the utilization of HIV testing services without decreasing the quality of care provided [45,46]. Last, the use of lay counsellors may lessen stigma and be an important connection to the community [42,45]. If the burden of counselling is reduced, the nurse counsellors may also have more time to keep themselves updated with the most recent PMTCT guidelines.

### Methodological limitations

A possible bias in this study was linked to the recruitment procedure. The nurses working in the selected clinics recruited the participants in the quantitative study. This may have made it difficult for the mother to decline taking part in the study, which may explain the extremely high participation rate (99.1%). Furthermore, it may have introduced a social desirability bias, where the mothers answered with what they assumed would be the right thing to say, rather than what they actually thought.

Additionally, recruitment from the clinics may have selected only mothers who had health-seeking behaviours. Due to the high regional immunization coverage [28], we do not believe that this introduced a bias. Due to our aim of comparing our results with those of four previous studies, the clinics included in the study were purposely selected. Since they were not randomly selected, the results may not be generalized.

The questions we asked about knowledge were all closed ended. It is important to differentiate between knowledge by recall, which implies active knowledge, and recognition of correct answers given to closed-ended options, which implies passive knowledge. Thus, the relatively high level of knowledge in our findings may be falsely high. However, we used open-ended questions in the qualitative interviews, and these findings largely supported the quantitative findings. Furthermore, the questions in the survey about condoms use as a preventive measure during pregnancy and birth may have been too simplified an approach to address a more complex issue.

The fact that the principal investigator, who also performed the fieldwork, was not conversant in the local language was a limitation to the qualitative data collection. The use of an interpreter may have created a distance between the interviewer and the interviewee and thereby moderated the quality of the interviews. Further, the principal investigator was unable to take active part in and moderate the FGDs. Efforts were made to decrease this limitation: the moderator was thoroughly informed about the topics of interest, and each FGD was transcribed and translated before the next FGD were performed so that subsequent adjustment could be made if necessary. The principal investigator analyzed qualitative data from transcripts that had been translated into English. To some extent, the translation process may have diluted some of the richness of the data.

Furthermore, observations of PMTCT counselling sessions were not performed at two of the participating clinics, which make us unable to fully compare the counselling received at the different clinics. Last, the concurrent mixed-method design did not allow for information gained by one method to inform the next method as it would have if a sequential design had been conducted.

## Conclusions

Routine counselling and testing for HIV at antenatal clinics was highly acceptable in this region. However, the counselling was suboptimal due to time and resource constraints. We interpret a higher level of PMTCT knowledge among the urban as opposed to the rural attendees as a result of differences in the start-up times of the PMTCT programme and hence programme maturation. Furthermore, as this study is the second conducted in this setting, we deduce that when the programme has had time to get firmly established, both its acceptance and the understanding of the topics dealt with during the counselling will increase.

## Competing interests

The authors declare that they have no competing interests.

## Authors' contributions

All authors participated in the design and planning of the study. The field work was conducted by EFF, supported by RM. The analysis and write up was carried out mainly by EFF, IMSE, MMdP and TT. All of the authors read and approved the final manuscript.

## Authors' information

EFF is a medical doctor and PhD candidate. She has research experience from a qualitative infant feeding study in Zambia. TT has a Masters in African Linguistics and is a paediatrician and professor at the Centre for International Health at University of Bergen, with extensive experience in health-related research in sub-Saharan Africa. MMdP is a nutritionist with a PhD in public health nutrition. She has extensive experience in mixed-methods research in Tanzania, South Africa and India. Findings from this current study were compared with her previous studies in the Kilimanjaro region. RM is a medical doctor with a PhD. She is working at the community health department in a hospital in Moshi, Kilimanjaro, region and has experience in conducting research in the region. IMSE is a medical doctor with a PhD in child health and nutrition and has experience in mixed-methods research in Uganda.
